# Construct validity of Advanced Practice Role Delineation tool: A confirmatory factor analysis

**DOI:** 10.1111/ijn.13064

**Published:** 2022-05-27

**Authors:** Krista Jokiniemi, Tarja Tervo‐Heikkinen, Jaana Peltokoski, Santtu Mikkonen

**Affiliations:** ^1^ Department of Nursing Science, Faculty of Health Sciences University of Eastern Finland Kuopio; ^2^ Clinical Development, Education and Research Centre of Nursing Kuopio University Hospital Kuopio Finland; ^3^ Central Finland Health Care District Jyväskylä Finland; ^4^ Department of Environmental and Biological Sciences and Department of Applied Physics University of Eastern Finland Kuopio Finland

**Keywords:** advanced practice nursing, factor analysis, nurse's role, psychometrics, statistical, survey

## Abstract

**Aim:**

To test the psychometric properties and latent structure of the modified Strong Model of Advanced Practice tool.

**Background:**

The Strong Model of Advanced Practice tool, developed in the United States in 1990s and its Australia modification, has been increasingly used to delineate nursing and advanced practice nursing roles. Few research‐driven efforts to develop and validate these tools have taken place.

**Design:**

Psychometric testing of the modified Strong Model of Advanced Practice tool.

**Methods:**

A confirmatory factor analysis was undertaken to examine the factors of the modified Strong Model of Advanced Practice tool and compare the model to the Australian Advanced Practice Role Delineation tool. The data were collected in September 2020.

**Results:**

The analysis of the data provided construct validity evidence of the underlying theoretical structures of the five‐factor modified Strong Model of Advanced Practice tool. The 45‐item modified Strong Model of Advanced Practice tool demonstrated satisfactory, slightly better psychometric properties and construct validity than the 40‐item Advanced Practice Role Delineation tool.

**Conclusions:**

Psychometric properties of the scale were evaluated and reported. Based on the statistical analysis, we suggest the use of the 45‐item modified Strong Model of Advanced Practice tool.

## Summary statement

What is already known about this topic?

- The Strong Model of Advanced Practice and its modifications have been increasingly used to delineate nursing and advanced practice nursing roles.
- Few research‐driven efforts to develop and validate these tools have taken place.

What this paper adds?

- The analysis of the data provides construct validity evidence of the underlying theoretical structures of the five‐factor modified Strong Model of Advanced Practice tool.
- The tool demonstrates satisfactory, slightly better psychometric properties and construct validity than the 40‐item Advanced Practice Role Delineation tool.

The implications of this paper:

- The validated modified Strong Model of Advanced Practice tool may be utilized to develop, standardize and delineate various nursing and advanced practice nursing roles within healthcare organizations.
- Based on statistical analysis, we recommend the use of a 45‐item Modified Strong Model of Advanced Practice tool.

## 1 INTRODUCTION

Countries are continuously developing clinical nursing and advanced practice nursing (APN) roles to respond to changing practice and healthcare demands. The clinical career ladder, from registered nurse (RN) to advanced practice nurse, has gained interest in several countries. Nurse managers, nursing associations and policy developers are required to consider the variability of nursing roles in human resource management. These premises highlight the need for validated tools to examine and distinguish the generalist, specialist and APN roles. The Advanced Practice Role Delineation (APRD) tool (Gardner et al., 2016), a modification of the Strong Model of Advanced Practice (SMAP) tool (Ackerman et al., 1996), has been increasingly used in several countries during the past few years. It has been found to be useful in the identification of advanced practice nursing activities and domains of practice. A recent content validation study resulted in the development of the 45‐item modified Strong Model of Advanced Practice (MoSMAP) tool in Finland. In this paper, we will report the findings of a construct validity study of this tool.

### 1.1 Background

Registered nurses (RNs), specialist nurses (SNs) and advanced practice nurses form a continuum of nursing roles according to the career structure from RN to advanced practice nurse. While RNs and SNs have diploma or bachelor level education and work at the generalist/specialist level, advanced practice nurses are post‐graduate educated, with extensive experience and advanced competency in the patient, clinical nursing leadership, organizational and scholarship spheres (Jokiniemi et al., 2019) Advanced practice nurses manage the care of complex populations by offering expert clinical care to patients, support the learning of nurses and interdisciplinary team, and facilitate innovation and health system outcomes according to their individual job descriptions (Fulton et al., 2019; Jokiniemi et al., 2020; Lewandowski & Adamle, 2009). Delineation of the role of the advanced practice nurses benefits the clarification of nursing roles, guides role descriptions, strengthens performance and role evaluations and informs the activities of optimal health system utilization (Jokiniemi et al., 2018).

The SMAP tool (Ackerman et al., 1996) was developed in the United States in the 1990s to differentiate and assess the level of APN activity by nurses in the areas of direct comprehensive care, support of systems, education, research, publication and professional leadership. An Australian research team developed the APRD tool, a 41‐item modification of the SMAP tool within the Australian context in the 2010s (Chang et al., 2010, 2012). The APRD tool has consequently been used by several researchers in several countries to investigate nursing and APN roles (Carryer et al., 2018; Gardner et al., 2016; Woo et al., 2019). In addition to the APRD tool, two further modifications of the SMAP tool exist: a 38‐item tool developed in Spain (Sevilla Guerra et al., 2018) and a 45‐item modification developed in the Finnish context.

Although the SMAP, APRD tool and their modifications have been used extensively in the past few years, there is limited research testing the content or construct of these tools. Within Australia, the APRD modification was developed based on a content (Chang et al., 2010) and a construct validation study (Chang et al., 2012). Based on these studies, one item (medical diagnosis) was omitted from the original tool; however, no new items were added to the tool. In Spain, as a result of a content validation study, a 38‐item modification of the APRD tool was developed and an additional construct validity study supported a six‐factor structure of the modified 38‐item tool (Sevilla Guerra et al., 2018). In Finland, the Australian 41‐item APRD tool content was explored by an expert panel to validate its content in the Finnish context in 2020. Based on the content validity study, one item (medical diagnosis) was omitted, and five new items were added, resulting in a 45‐item MoSMAP tool. Table 1 summarizes the studies validating the SMAP tool and its international modifications.

**TABLE 1:** Validation studies of Strong Model of Advanced Practice tool and its modifications

Research method, authors, year	Sample	Subscales, items, Cronbach alphas for subscales	Tool psychometric tests
The US – 42 item Strong Model of Advanced Practice			
Tool development. Ackerman et al., 1996	Literature	Direct comprehensive care (15 items) Support of systems (9 items) Education (6 items) Research (6 items) Publication and professional leadership (6 items)	‐
Descriptive, exploratory pilot study. Mick & Ackerman, 2000	*n* = 18	Same subscales	Descriptive mean practice activity scores among clinical nurse specialists and nurse practitioners.
Australia – 41‐item APRD tool (modification from Strong Model of Advanced Practice)			
Content validation study. Chang et al., 2010	*n* = 16, 15, 15	Direct comprehensive care (14 items) Support of systems (9 items) Education (6 items) Research (6 items) Publication and professional leadership (7 items)	All but 2 items reached 75% cut‐off point. One item was left out of the survey (1.3 make a medical diagnosis) and one item was divided into two (4.6)
Construct validity study utilizing the original Strong Model of Advanced Practice (EFA, principal axis factoring) Chang et al., 2012	*n* = 658	Direct comprehensive care (16 items) (α = 0.95) Support of systems (9 items) (α = 0.93) Education (4 items) (α = 0.83) Research (6 items) (α = 0.90) Publication and professional leadership (6 items) (α = 0.94).	Based on EFA ‘make a medical diagnosis’ was omitted. Five factors, 41‐items, accounted for just over 70% of the total variance. Cronbach alpha: 0.94
Finland – 45‐item Finnish APRD tool (modification of Australian 41‐item APRD tool)			
Content validation study.	*n* = 9, 8, 5	Direct comprehensive care (14 items) Support of systems (11 items) Education (8 items) Research (6 items) Publication and professional leadership (6 items)	Item, make a medical diagnosis, was omitted and 5 new items added. The item content validity index varied between 0.88 and 1.00 and the scale content validity index average was 0.97.
Cross‐sectional study. Jokiniemi et al., 2021	*n* = 1497	APRD Direct comprehensive care (14 items) (α = 0.90) Support of systems (8 items) (α = 0.87) Education (6 items) (α = 0.76) Research (6 items) (α = 0.84) Publication and professional leadership (6 items) (α = 0.89) MoSMAP Direct comprehensive care (15 items) (α = 0.90) Support of systems (11 items) (α = 0.92) Education (7 items) (α = 0.85) Research (6 items) (α = 0.84) Publication and professional leadership (6 items) (α = 0.89)	Cronbach's alpha score of a coefficient for the 40‐item APRD‐tool was 0.90, and for the modified 45‐item tool 0.92.
Spain – 38‐item Spanish APRD tool (modification of Australian 41‐item APRD tool). Guerra et al., 2018			
Construct validity study. Sevilla Guerra et al., 2018	*n* = 151	Expert care planning (7 items) (α = 0.92) Integrated care (8 items) (α = 0.91) Interprofessional collaboration (6 items) (α = 0.84) Education (6 items) (α = 0.83) Research and evidence‐based practice (6 items) (α = 0.82) Professional leadership (5 items) (α = 0.86)	EFA: 6 factors accounted for 65.8% of variance. Cronbach alpha = 0.86 Test–retest stability (n = 123) = all items showed Temporal stability (*P* < .05)
Content validation study. Sevilla Guerra et al., 2018	*n* = 39	Same	S‐CVI = 0.95
Construct validity (CFA), Sevilla Guerra et al., 2018		Same	CFA = the hypothesised 6‐factor model had a better fit than the 5‐factor model: χ^2^/*df* = 1.97 vs. 2.17; CFI = 0.79 vs. 0.74; TLI = 0.77 vs. 0.72; SRMR = 0.10 vs. 0.12

Abbreviations: APRD, Advanced Practice Role Delineation; CFA, confirmatory factor analysis; CFI, comparative fit index; EFA, exploratory factor analysis; MoSMAP, modified smart model of advanced practice; S‐CVI, scale content validity index; SRMR, standardized root‐mean‐square residual; TLI, Tucker–Lewis index.

The increased use of the SMAP and APRD tools in recent years highlights a global need for a valid tool to differentiate and examine the role activities of advanced practice nurses. Within this study, we tested and reported construct validity research to examine the 45‐item Finnish MoSMAP tool. Confirmatory factor analysis (CFA) was used to establish the best‐fitting model to the proposed 45‐item, five‐factor model. A comparison for the 41‐item, five‐factor APRD tool (Gardner et al., 2016) was also conducted.

## 2 METHODS

### 2.1 Aim

The aim of this study was to conduct a CFA test on the MoSMAP tool to test the psychometric properties and latent structure of the tool. The items within the MoSMAP scale are hypothesised to have at least modest inter‐correlations, and the tool should measure several distinct qualities with clustering corresponding to the five subscales (APN domains) (Ackerman et al., 1996) of direct comprehensive care: support of systems, education, research, publication and professional leadership (Prudon, 2014).

### 2.2 Design

A psychometric study with an online, 45‐item MoSMAP tool was trialled using CFA to verify the number of underlying dimensions of the instrument (factors) and the pattern of item–factor relationships (factor loadings) (Brown, 2015).

### 2.3 Participants

A census sample of six Finnish healthcare district nursing personnel was recruited through the organizations' contact persons. To be eligible for this study, the participants had to be (a) a RN/midwife, (b) a SN or (c) an advanced practice nurse. In total, 1497 responses were analysed in this study.

### 2.4 Data collection

Participant recruitment and data collection were conducted in September 2020. Data were collected using an online self‐report questionnaire. The 45‐item MoSMAP measured the nurses' activities during a typical month on a 5‐point Likert‐type scale (0, *never*; 1, *rarely*; 2, *sometimes*; 3, *often*; and 4, *always*). Five role domains of direct comprehensive care (support of systems, education, research, publication and professional leadership) form the organizing framework of the scale (Ackerman et al., 1996).

### 2.5 Validity and reliability

#### 2.5.1 The tool and its translation

A 45‐item MoSMAP tool, which contains the 41‐item Australian APRD tool (excluding the item ‘makes a medical diagnosis’), was used in this study. The 41‐item APRD tool is described in detail by Gardner et al. (2016). Initially, the 41‐item APRD tool was translated from English to Finnish and back‐translated from Finnish to English by two independent authorized translators of a professional translation service in 2019. The translation and back‐translation were assessed item by item by team members from the research field of nursing and APN roles who were fluent in Finnish and English. The meaning, accuracy, wording and grammar of the tool were assessed, and clarifications were suggested as needed (Squires et al., 2013). After translation, the 41‐item tool was subjected to a content validity examination.

#### 2.5.2 Tool content validation

A content validation process was conducted between September–October 2019 to validate the content of the tool in Finnish context. During three iterative rounds (*n* = 9, *n* = 8 and *n* = 5), the panel of experts in the areas of advanced practice nursing, healthcare management and APN education evaluated the 41‐item APRD tool items in terms of their content and relevancy to nursing practice. During this process, five missing items were added to the tool, of which three were assigned to the domain of support of systems (involving the integration of evidence‐informed practice procedures, promotion of the innovation activity within the practice area and facilitation of the implementation of change) and two items to the education domain (involving the development and conduction of staff education programs and evaluation of nursing staff competence). One item, ‘makes a medical diagnosis’, was removed from the tool due to a low item content validity index score (I‐CVI) of 0.4. Based on the content validation process, a 45‐item modification of the APRD tool, MoSMAP, was developed. The developed tool received a high‐scale average CVI of 0.97; thus, the criteria may be regarded as valid in terms of content in the Finnish context.

#### 2.5.3 Pilot testing of the tool

The surveys were piloted with participants who met the inclusion criteria of the study (*n* = 5). Feedback was requested regarding the clarity and understanding of the language, survey functionality and time burden, as well as appropriateness of the length of the survey.

### 2.6 Data analysis

The Statistical Package for the Social Sciences (SPSS, 2017) version 25 and IBM SPSS Amos 27 were employed to conduct CFA and calculate α coefficients. The sample size of 1497 in our study was adequate for CFA (Kline, 2015). In addition, data were missing completely at random (*P* > 0.05) based on Littles' Missing Completely at Random (MCAR) test (Allison, 2002). No data was imputed, as with the Amos program, as analysis can be performed with missing data (Arbuckle, 2016). The correlations between the items and predicted scales were examined, and several descriptive fit parameters (comparative fit index [CFI], Tucker–Lewis index [TLI], root mean square error of approximation [RMSEA], and minimum discrepancy function divided by degrees of freedom [CMIN/DF]) were used to evaluate the final model. A cut‐off value close to 0.90 for TLI and CFI and a cut‐off value under 0.06 for RMSEA (to evaluate the model's general adequacy) were considered a good fit of the data (Brown, 2015).

### 2.7 Ethical considerations

The ethical aspects of the research were evaluated by the University of Eastern Finland Committee on Research Ethics with a supportive statement for the research (statement number 22/2018). Each participating organization granted research permission to administer the questionnaire. Prospective participants were sent an information sheet about the study with a link to the survey via their hospital contact person. Participation in the study was voluntary, anonymous, and could be discontinued at any time. The participants were informed that completion of the e‐survey was regarded as implied consent (TENK, 2019; World Medical Association, 2013).

## 3 RESULTS

### 3.1 Sample

A total of 1512 nurses returned the self‐reported survey, yielding an overall response rate of 10%. A total of 1497 responses were included in the data analysis: 1241 RNs, 90 registered midwives (RM), 61 SN, 53 clinical nurse specialists (CNS) and 13 clinical teachers in nursing (CTN). A majority of the participants worked at a university hospital setting (95%). Eighty‐six per cent had undergraduate degrees, while 13% had master's or doctoral degrees. Most respondents were female with mean age of 41.5 years. A detailed description of the sample may be found in the study by Jokiniemi et al. (2021).

### 3.2 Construct of the modified Strong Model of Advanced Practice

In the 45‐item MoSMAP tool, a five‐factor model was retained for the final CFA solution. The tool factors included the same items as the 41‐item APRD tool, with the exception of the removal of item ‘makes a medical diagnosis’. Based on the modelling, one item, 3.6. ‘provide appropriate patient and family education’, was moved to factor 1 (domain 1), direct comprehensive care. The item shift was also supported by an exploratory factor analysis item factor loading, which were checked for consistency with the CFA results.

### 3.3 Confirmatory factor analysis for the modified Strong Model of Advanced Practice

**FIGURE 1:**
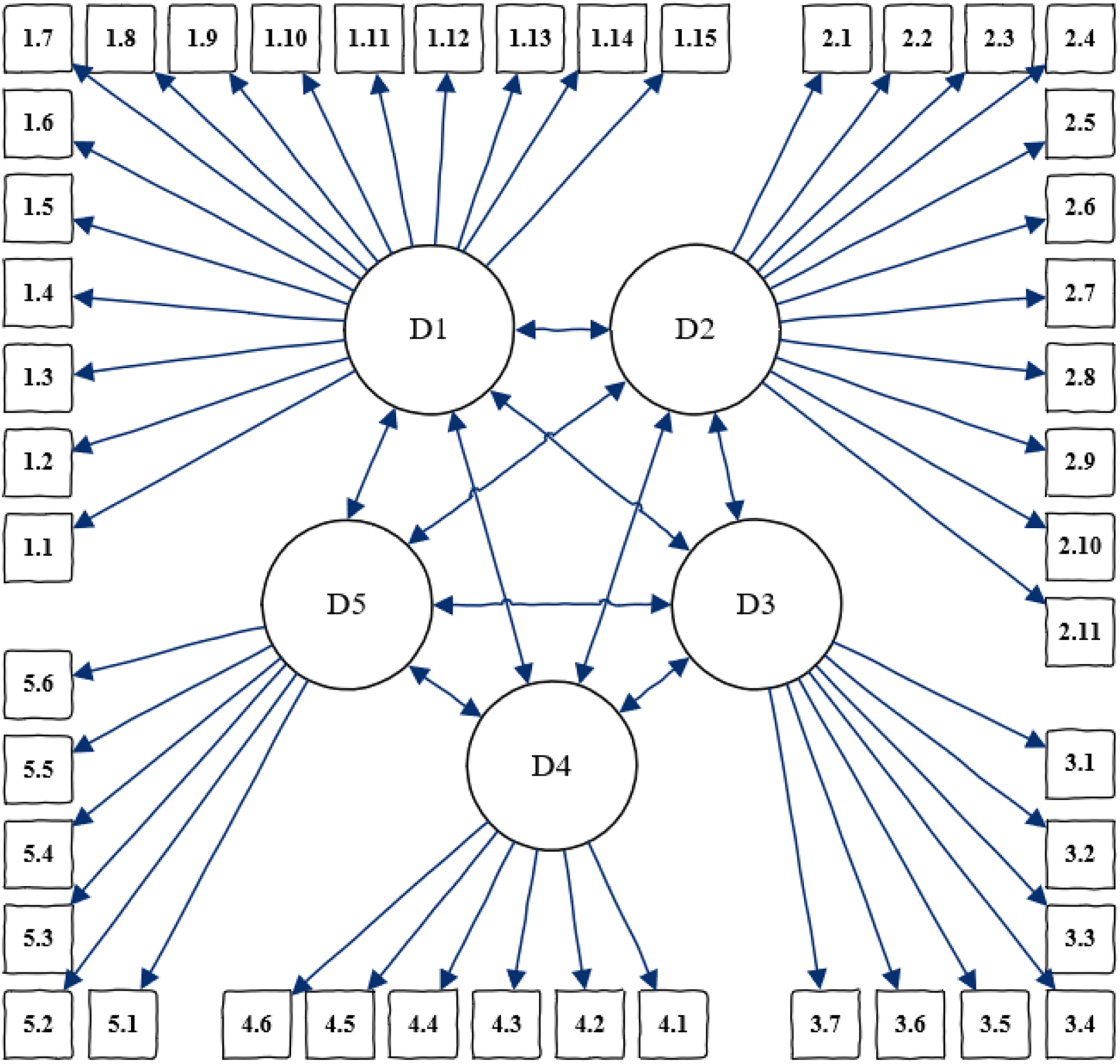
Confirmatory factor analysis of modified Strong Model of Advanced Practice. Abbreviation: D, domain

The model was specified based on a theoretical model developed for CFA. The results are reported with standardized regression coefficients of the items related to the latent variables. The model is presented in graphic form (Figure 1).

The MoSMAP model indices proved to be satisfactory. The range of the standardized factor loadings were moderate to high (0.252–0.820), supporting convergent validity. Factor covariances may be seen in Table 2. For item regression weights, please see supporting information (Table S1). A CFI of 0.907 and TLI of 0.892 were considered very good, and a CMIN/DF score of 5.08 and RMSEA value of 0.52 were both considered good (Brown, 2015).

**TABLE 2:** Factor covariances

	Factor covariances	SE	*p*
F1–F2	−.064	.018	***
F1–F3	−.098	.019	***
F1–F4	−.065	.012	***
F1–F5	−.054	.012	***
F2–F3	.644	.034	***
F2–F4	.392	.022	***
F2–F5	.330	.020	***
F3–F4	.393	.021	***
F3–F5	.352	.020	***
F4–F5	.247	.014	***

Abbreviations: F, factor; SE, standard error. ***p < .001.

The 41‐item APRD model was also applied. Model fit was quite good; however, the MoSMAP model indices proved satisfactory with slightly stronger values than the 40‐item tool, indicating that the 45‐item tool fits better for our data.

## 4 DISCUSSION

In this article, we reported on research validating the construct of the MoSMAP tool. The SMAP and APRD tools have increasingly been used to examine and distinguish nursing and APN roles. Research on their content and construct aids in the evaluation of its psychometric properties and ability to measure advanced practice nursing activities. Due to the content validation of the tool, it may better reflect the contemporary healthcare environment with the items added to support of systems (*n* = 3) and education domain (*n* = 2). Similar to some other countries, the item ‘makes a medical diagnosis’ was removed from the MoSMAP tool due to the item not being in the scope of nurses' practice (Chang et al., 2010). However, if needed, it may be re‐added to the tool.

Overall, the fit statistics suggest that the estimate model reproduces the sample covariance matrix reasonably well; thus, the evidence suggest a good construct validity. Therefore, we can be fairly confident that the model domains behaved as they should, in terms of the unidimensionality of the five measures and the way the construct related to the other measures. Empirically found item clustering supports the content validity of the items and corresponds to the construction of the tool (see Prudon, 2014). The results of our study support the convergent validity of the model. Both the APRD and MoSMAP models were evaluated to fit well with slightly better construct validity and psychometric property values in the 45‐item MoSMAP tool. Therefore, the 45‐item model may be recommended for use in future studies to further examine model results beyond this study. Cronbach's alpha coefficient for the modified overall tool of 0.92 indicates adequate reliability (Jokiniemi et al., 2021).

Despite the increased development of APN roles and the utilization of the SMAP or APRD tools to guide this work, there exists only limited research on the validation of the content or construct of these tools. In addition to our research, we are aware of only one previous study (Sevilla Guerra et al., 2018) investigating the construct of the SMAP or APRD tool utilizing CFA. Sevilla Guerra and colleagues conducted comprehensive translation, adaptation and psychometric testing of the APRD tool between 2015 and 2016. Based on their exploratory factor analysis (EFA), a six‐factor structure was suggested and although the subsequent CFA suggested that both models were adequately fitted to the data, the hypothesised six‐factor model had a better fit than the five‐factor model. The limitation of the Spanish study, however, was the low sample size of 151 due to low numbers of APNs in the country at the time of the study. A higher sample size would have improved the model fit tests (Sevilla Guerra et al., 2018). As the empirical and conceptual foundations of the SMAP/APRD (which have only minor differences) are getting stronger due to increased use and research, we emphasize the use of CFA in future studies to guide the specification and evaluation of the factor models used. EFA is typically used earlier in the process of scale development and construct validation, whereas CFA is used in later phases to confirm the underlying structure established with prior empirical (EFA) and theoretical grounds. As there are specifications made in regard to the number of latent factors and the pattern of relationships between them within the SMAP and APRD, CFA can provide compelling evidence of the convergent and discriminant validity of these theoretical constructs (Brown, 2015).

We have conducted a comprehensive content and construct validity testing on the APRD tool and developed a MoSMAP, a 45‐item contemporary modification of the APRD tool. In the future, it is important to test the MoSMAP tool in other contexts and culturally diverse healthcare settings to further validate its construct beyond the present study. The tool may be used to guide nursing and APN roles and clinical career ladder development, as well as role standardization. In addition, it may inform nurse managers by offering means to delineate various nursing and advanced practice nursing roles within healthcare organizations.

### 4.1 Limitations

The participants of this study were only from Finland, where APN roles have existed for approximately two decades. Although the study had a low response rate, which is typical when e‐surveys are involved and with the current coronavirus disease 2019 (COVID‐19) situation, we evaluated the sample to be representative of the target nursing population, and adequate for CFA. In the future, further testing of the scale is needed to examine the scale in wider contexts; thus, results with more culturally diverse samples may differ from the current sample. Therefore, examination of the scale properties in new samples is important.

## 5 CONCLUSION

Testing of the theoretical construct of an existing tool gives valid information about its usefulness in nursing practice. Tested tools are needed to develop nursing science and ensure the validity of the tools we use to study the practice of nursing. The findings of this study provided evidence that support the reliability and validity of the 45‐item MoSMAP tool. This tool may be helpful for nursing managers and researchers in identifying the activities of generalist, specialist, and APN roles with respect to the five domains of practice. The 45‐item MoSMAP tool demonstrated satisfactory, slightly better psychometric properties and construct validity than the 40‐item tool. Further validity tests should be conducted in subsequent studies to further examine its construct structure on samples of nurses in different cultures and contexts. The results are regarded as relevant to the international APN community, in informing the validity of available tools to guide and delineate nursing and APN role development.

## CONFLICT OF INTEREST

No conflicts of interest have been declared by the authors.

## AUTHORSHIP STATEMENT

KJ, TT‐H, JP and SM made substantial contributions to conception and design, or acquisition of data, or analysis and interpretation of data. KJ, TT‐H, JP and SM involved in drafting the manuscript or revising it critically for important intellectual content. KJ, TT‐H JP and SM give final approval of the version to be published. Each author should have participated sufficiently in the work to take public responsibility for appropriate portions of the content. KJ, TT‐H, JP and SM agreed to be accountable for all aspects of the work in ensuring that questions related to the accuracy or integrity of any part of the work are appropriately investigated and resolved.

## Supporting information


**Table S1** Item regression weights

## Data Availability

Research data are not shared.
