# Supplementary material for: Construct validity of Advanced Practice Role Delineation tool: A confirmatory factor analysis
Source: Int J Nurs Pract. 2022 May 27;28(5):e13064. doi: 10.1111/ijn.13064 (PMC9788128; doi:10.1111/ijn.13064)
Supplement: Supplementary file 1 — Table S1 Item regression weights [file IJN-28-e13064-s001.docx]

Supplemental file 1

Table 3: Item Regression Weights

|  | **Regression Weights** | **S.E.** | **Standardized Regression Weights** | **p** |
| --- | --- | --- | --- | --- |
| D1 | 1,000 | - | ,599 | *** |
| D2 | ,956 | ,041 | ,662 | *** |
| D3 | 1,152 | ,057 | ,659 | *** |
| D4 | 1,302 | ,058 | ,764 | *** |
| D5 | ,728 | ,042 | ,529 | *** |
| D6 | 1,390 | ,066 | ,754 | *** |
| D7 | 1,349 | ,059 | ,775 | *** |
| D8 | 1,224 | ,054 | ,764 | *** |
| D9 | ,743 | ,038 | ,609 | *** |
| D10 | ,410 | ,046 | ,525 | *** |
| D11 | ,867 | ,048 | ,562 | *** |
| D12 | 1,155 | ,056 | ,674 | *** |
| D13 | .838 | ,046 | ,561 | *** |
| D14 | .893 | ,048 | ,585 | *** |
| S15 | 1.000 | - | ,704 | *** |
| S16 | ,700 | ,030 | ,639 | *** |
| S17 | ,748 | ,028 | ,731 | *** |
| S18 | ,833 | ,030 | ,764 | *** |
| S19 | 1,040 | ,035 | ,815 | *** |
| S20 | ,907 | ,030 | ,820 | *** |
| S21 | ,999 | ,034 | ,797 | *** |
| S22 | ,693 | ,036 | ,527 | *** |
| S23 | ,907 | ,036 | ,703 | *** |
| S24 | ,889 | ,038 | ,641 | *** |
| S25 | ,714 | ,040 | ,486 | *** |

Table 3 continues….

…Table 3 continues

| E26 | ,821 | ,026 | ,775 | *** |
| --- | --- | --- | --- | --- |
| E27 | ,586 | ,037 | ,426 | *** |
| E28 | ,551 | ,023 | ,610 | *** |
| E29 | ,630 | ,031 | ,469 | *** |
| E30 | 1,000 | - | ,801 | *** |
| E31 | ,505 | ,042 | ,333 | *** |
| E32 | ,994 | ,024 | ,810 | *** |
| E33 | ,805 | ,026 | ,755 | *** |
| R35 | ,911 | ,036 | ,665 | *** |
| R36 | ,363 | ,017 | ,560 | *** |
| R37 | 1,349 | ,049 | ,757 | *** |
| R38 | 1,213 | ,033 | ,806 | *** |
| R39 | 1,000 | - | ,807 | *** |
| P40 | 1,000 | - | ,759 | *** |
| P41 | ,882 | ,033 | ,696 | *** |
| P42 | 1,045 | ,034 | ,788 | *** |
| P43 | ,670 | ,022 | ,795 | *** |
| P44 | ,956 | ,032 | ,771 | *** |
| P45 | .868 | ,031 | ,738 | *** |

D=direct comprehensive care, S=support of systems, E=education, R=research, P=publication, and professional leadership, S.E.=standard error
